# Interconnected Developmental Trajectories of the Brain, Gut, and Sleep in Early Life: The First 1000 Days of Nutritional Opportunity

**DOI:** 10.3390/nu18030445

**Published:** 2026-01-29

**Authors:** Devyani Chaturvedi, Shikha Snigdha, Michael A. Grandner, Nicole Avena, Punam Patel

**Affiliations:** 1 SmartyPants Vitamins, Unilever Wellbeing Collective, El Segundo, CA 90245, USA; 2 Unilever Wellbeing Collective, San Francisco, CA 94102, USA; shikha.snigdha@unilever.com (S.S.); punam.patel@unilever.com (P.P.); 3 Department of Psychiatry, University of Arizona, Tucson, AZ 85714, USA; grandner@gmail.com; 4 Department of Neuroscience, Icahn School of Medicine at Mount Sinai, New York, NY 10029, USA; nicole.avena@mssm.edu

**Keywords:** children, infants, gut, brain, sleep, neurodevelopment, nutrition, HMO, probiotics, DHA

## Abstract

The first 1000 days of life, from conception through the second year, represents a uniquely sensitive period for neurodevelopment. During this time, multiple physiological systems undergo rapid and coordinated maturation. Among these, the brain, gut, and sleep system form a tightly interconnected triad, exerting reciprocal influences on each other and playing a pivotal role in shaping lifelong cognitive, emotional, and behavioral trajectories. Disruptions in any one of these domains can reverberate across the others, amplifying developmental vulnerabilities. A key modifiable factor that can modulate this gut–brain–sleep triad is nutrition. In this review, we synthesize current evidence on the interconnected development of the brain, gut, and sleep systems and examine the role of key nutrients in shaping these pathways. We also identify critical gaps in the literature and highlight opportunities for future research to better understand how early-life nutritional interventions can optimize neurodevelopmental outcomes.

## 1. Introduction

The adult brain is a complex structure with more than 100 billion neurons [1]. The 3-mm neural tube’s transformation into a fully functional brain represents a rapid phase of growth and development [2]. Most of these processes begin within the initial 1000 days of life, laying the foundation for brain development and later cognitive growth [3]. While intrinsic genetic factors drive much of this neurodevelopment, emerging evidence highlights the influence of extrinsic factors, particularly gut health, sleep architecture, and nutrition [4,5]. In early postnatal life, the infant gut microbiota is shaped by prenatal, perinatal, and postnatal exposures and typically stabilizes around 2–3 years of age [6]. However, disruptions in this process may result from factors such as cesarean delivery, antibiotic exposure, early-life stress, or diets lacking key nutrients and probiotics that favorably modulate the gut microbiota, thereby directly impacting neurodevelopmental outcomes. The gut microbial community and the developing brain evolve in parallel, with increasing evidence suggesting a bidirectional relationship that influences emotional regulation and cognitive function [7]. The gut also appears to modulate sleep and vice versa, another foundational component of healthy neurodevelopment [8]. In infants and young children, sleep is essential for synaptic pruning, memory consolidation, attention, executive functioning, and emotional control. Disrupted sleep during this critical period may adversely affect long-term brain health [9].

Among the many influences on early development, nutrition stands out as the most modifiable factor, shaping the brain, gut, and sleep. Beyond neural development, early-life nutrition also shapes the gut microbiome, which is discussed in detail later. Adequate nutrition during the first 1000 days supports healthy gut microbiota, the cornerstone of long-term immune, metabolic, and neurodevelopmental health. Finally, strong evidence is emerging for a two-way relationship between nutrition and sleep during this phase of life. Diet quality and specific nutrients modulate hormonal pathways that regulate sleep, which, in turn, influences total energy intake and food choices through biological and behavioral mechanisms [10,11].

## 2. Development of Brain: First 1000 Days

The maturation of the neural tube into a functional brain exemplifies the remarkable pace and intricacy of early human developmental processes [2]. It starts during the 3rd and 4th week of gestation [12]. Even at this early stage, nutrition plays an instrumental role in supporting the development and differentiation of various organs. The rapidly developing embryo relies on the mother’s nutrient stores to fuel its growth [13]. Nutrients such as folic acid, choline and iron are especially critical during this phase. Research shows that folic acid is essential for proper neural tube closure, and its deficiency can lead to neural tube defects (NTDs), which may impair cognitive development [14]. Similarly, complications such as preterm birth, low birth weight, small-for-gestational-age (SGA), and an increased risk of neurodevelopmental disorders (NDDs) in children are few of the adverse birth outcomes associated with low iron status during pregnancy [15,16].

Physical and mental stressors experienced by the mother during this period, and in the later months can also impact fetal development by activating the hypothalamic–pituitary–adrenal (HPA) axis. This leads to increased cortisol production, which crosses the placenta and exposes the fetus to maternal stress signals [17]. Such exposure can disrupt fetal brain development and may result in lasting cognitive and neuropsychological consequences in the offspring [18]. Supporting this, a study conducted with pregnant women carrying single fetuses between 28 and 36 weeks of gestation found that the emotions experienced by the mothers influenced their babies’ movements. When the mothers watched a joyful film, an increase in fetal activity was observed, whereas viewing a sad film led to a decrease in fetal movements. These findings indicate a connection between the mother’s emotional state, stress levels, and the fetus’s behavioral responses [19].

Overall, this is a time during which the brain displays remarkable plasticity, allowing for significant modification and refinement of synaptic connections. This period serves as a critical window of opportunity, where various mechanisms can shape neural development and ultimately neurodevelopmental outcomes.

## 3. Development of Gut Microbiota: The First 1000 Days

**Figure 1:**
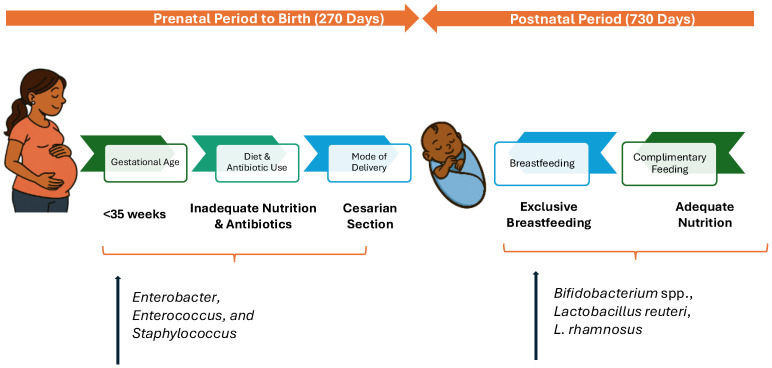
Factors Impacting Gut Microbiota in the First 1000 Days. In the prenatal to early childhood period, gut microbiota development is shaped by factors such as gestational age, mode of delivery, antibiotic exposure, breastfeeding, and timing of solid food introduction. Premature birth and C-section delivery can disrupt microbial diversity and increase infection risk, while breastfeeding and exposure to HMOs promote beneficial bacteria and support immune and neurodevelopment.

As with the developing brain, the gut microbiome undergoes rapid maturation during the first 1000 days of life, a critical window for establishing its composition and functional capacity with lasting implications for metabolic, immune, and neurodevelopmental outcomes [20]. Gut microbes also play a key role in influencing neural development, modulating neurotransmitter systems, and impacting behavior [21,22], making gut–brain interactions particularly consequential during the perinatal period and the first two years of postnatal life (Figure 1). Disruptions during these windows can negatively impact neurodevelopmental and neuropsychological outcomes [23].

In the first two years after birth, the gut microbiota develops rapidly from a low-diversity state to a more complex composition. Colonization begins at birth [24] and is influenced by several factors discussed below. By 2–3 years of age, it typically resembles a more stable, adult-like state [25].

### Factors Impacting Gut Microbiota in the First 1000 Days

**Gestational age:** Gestational age at birth is a critical factor affecting diversity of the gut microbiome of an infant. Infants born prematurely frequently possess an underdeveloped gastrointestinal system, which can create a harmful cycle where both the immature immune system and gut lining contribute to widespread inflammation or sepsis. This may necessitate use of antibiotics, thereby promoting a reduction in diversity of gut bacterial populations and an increase in harmful microorganisms [26]. The meconium of infants born prematurely (<35 weeks gestation) has been shown to contain a gut microbiota characterized by a reduced number of Bifidobacteria, key bacteria responsible for breaking down human milk oligosaccharides (HMOs) for energy and promoting immune development, and increased number of *Enterobacter*, *Enterococcus*, and *Staphylococcus*. This microbial imbalance heightens vulnerability to infections and disrupts healthy microbiome development compared with term infants [25,27].

**Mode of delivery at birth:** Several studies have noted that the mode of delivery significantly influences the early infant gut microbiome. Infants born via cesarean section (C-section) demonstrate differences in their microbiota compared to those born through vaginal delivery. More specifically an increased presence of *Bifidobacterium* spp., *Lactobacillus reuteri*, *Lactobacillus rhamnosus* and reduction in opportunistic pathogens such as *Enterococcus* and *Klebsiella* spp. is observed in vaginally delivered infants [28,29]. The oral and nasal cavity and other exposed parts of an infant mimic the microflora of the maternal vagina, while that of C-section delivered infants is similar to that of mothers’ skin surface, dominated by *Propionibacterium*, *Corynebacterium*, and *Staphylococcus* spp. [30]. This may heighten susceptibility to opportunistic infections. Given the critical role of C-section in safeguarding maternal and neonatal outcomes in high-risk pregnancies, attention to interventions such as supplementation with probiotics [31] and breastfeeding should be encouraged.

**Exposure to antibiotics:** Antibiotics are often administered during the cesarean birth process, further altering the gut microbiome of both the mother and the infant. Exposure to antibiotics during pregnancy is extremely common [32], typically for several reasons such as premature labor, intrapartum fever during labor, and to reduce the risk of neonatal Group B Streptococcus infection. Antibiotics are used by around 70% of pregnant women at some point during pregnancy and represent close to 80% of all prescription drugs given to pregnant individuals [32]. Unfortunately, antibiotic use can lead to decreased microbial diversity, alterations in the functional characteristics of the gut microbiota, and the emergence and proliferation of antibiotic-resistant strains, which increase the host’s vulnerability to pathogenic infections. These changes in the microbial community may last for as long as 12 weeks following treatment [33]. Similarly to C-section delivery, in cases where antibiotics are unavoidable, supplementation with probiotics may be a viable method to circumvent the microbiome disruption that may occur.

**Breastfeeding:** Breastfeeding exerts major influence on the microbiome composition [34]. An infant’s gut microbiota is affected through both direct exposure to the mother’s milk microbiota and indirectly via bioactive components in milk such as HMOs, SIgA, and antimicrobial compounds that regulate microbial proliferation and function [35,36]. For instance, a study of 91 term infants that were either exclusively breast-fed or formula-fed, showed increase Bifidobacterium and Bacteroides and decreased Streptococcus and Enterococcus levels in the breast-fed group compared to the formula fed group [37]. Studies in the past few years have also increasingly reported significant associations between exposure to HMOs in particular during the breastfeeding process and neurodevelopmental outcomes in infants [36]. In a randomized, double-blind trial, comparing formula-fed vs. breastfed infants, inclusion of HMOs in the formula led to increased systemic levels of microbial-derived secondary bile acids, bringing those metabolite levels into alignment with those observed in breastfed infants [38]. Overall, these findings indicate that the recovery of specific metabolites produced through gastrointestinal microbial activity may be linked to the presence of HMOs. In addition, both the timing and nature of complementary food introduction play key roles in shaping gut microbial composition [39]. Introduction of solid foods, i.e., at or earlier than 3 months of age can lead to changes in the levels of gut bacteria and bacterial byproducts. When infants are introduced to foods that are rich in complex carbohydrates, their gut microbiome tends to rapidly mimic an adult’s gut microbiome. Understanding the evolution of bacterial composition in the human gut from infancy and the impact of feeding modality and introduction of solids may help in the development of strategies to support early establishment of health-promoting microbiota. This may in turn have physiological benefits that last through the lifespan [40].

**Bi-directional Relationship of Gut–Brain:** The impact of gut microbiome on the nervous system (both central and enteric) has been demonstrated in several rodent studies. The hypothesis of gut microbiome’s influence on brain was first demonstrated by Heitz et al. in 2011 where the authors showed that germ-free mice displayed increased motor activity (often associated with increased risk-taking behaviors that suggests impaired cognitive function), compared with specific-pathogen-free (SPF) mice with a normal gut microbiota [41]. In a follow-up study, fecal microbiota from young (3–4 months) or aged (19–20 months) donor mice were transplanted into older recipient mice of similar age (19–20 months). The results demonstrated that receiving microbiota from young donors reversed aging-related alterations in immune function both systemically and in the brain, alongside restoring changes in the hippocampal metabolomic and transcriptomic profiles. Moreover, the young donor microbiota alleviated specific cognitive declines associated with aging in the recipient mice [42]. This bidirectional impact is observed not just on the central nervous system (CNS) but also on the enteric nervous system (ENS). For instance, germ-free (GF) mice demonstrate a reduction in the number of enteric neurons along with a concurrent deficit in gut motility. This effect has also shown to be reversed when the microbiota is reconstituted in these mice [43]. Such studies, along with the colonization of the microbiome coinciding with the development of the nervous system in a coordinated manner [44], clearly demonstrate the interdependence of a healthy microbiome of brain health and cognitive function.

**Figure 2:**
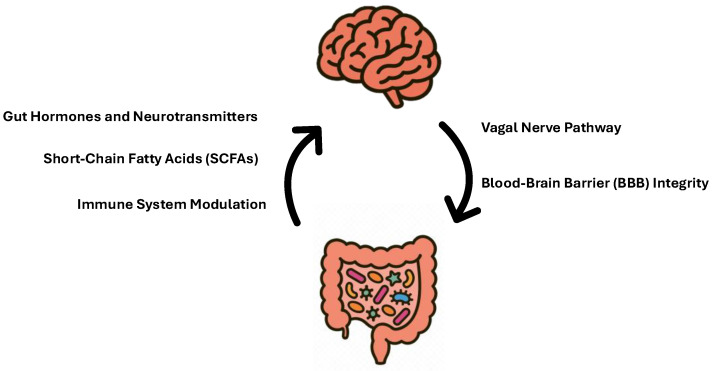
Bidirectional Gut–Brain Communication Pathways. The gut microbiome and brain are deeply interconnected, influencing each other through neural, hormonal, immune, and metabolic pathways. Key mechanisms include the vagus nerve, neurotransmitter production, short-chain fatty acids, immune modulation, and blood–brain barrier integrity.

However, environmental disruptions during prenatal life including nutritional stressors can interfere with this early developmental process, weakening the connection and leading to both clinical and subclinical outcomes, from altered stress responses to a range of NDDs [21,45]. These effects are mediated via several different pathways including neural pathways (vagus nerve), endocrine impact, immune molecules, and humoral links (cytokines, short-chain and long-chain fatty acids) [46,47,48] shown in Figure 2.

a. **Vagal Nerve Pathway**

As a key part of the microbiota–gut–brain axis, the vagus nerve facilitates two-way communication between the gut microbiota and the brain, carrying roughly 80% afferent signals (gut to brain) and 20% efferent signals (brain to gut). It does not physically connect with the microbiota but responds to signals transmitted via microbial metabolites, inflammation, or neuroendocrine cells influenced by the gut microbial ecosystem [49,50]. Certain neurotoxic metabolites generated by the gut microbiota can stimulate the vagal nerve and may impair brain function, disrupt sleep [51], and alter stress responses [52]. To this effect, animal studies have demonstrated that treatment with *Lactobacillus rhamnosus* can reduce stress-induced corticosterone levels and alleviate anxiety and depression-like behaviors in rats [53]. Additionally, the neuroendocrine pathway serves as a key mechanism through which the gut microbiome modulates the central nervous system and the HPA axis, primarily byregulation of serotonin (5-HT), cortisol, and melatonin secretion [5]. Microbiota-driven regulation of serotonin (5-HT) synthesis occurs through modulation of tryptophan hydroxylase (TPH) activity in enterochromaffin cells, with germ-free models demonstrating substantially reduced 5-HT availability and altered diurnal signaling to vagal–hypothalamic sleep and metabolic pathways. Several gut-associated taxa, including *Lactobacillus*, *Lactococcus*, *Prevotella*, *Streptococcus thermophilus*, *Escherichia coli*, *K-12*, *Morganella morganii*, *Klebsiella pneumoniae* and *Staphylococcus aureus*, can generate 5-HT and biogenic amines that are subsequently converted into melatonin, thereby linking microbial metabolism to circadian rhythm regulation and stress-axis integration via cortisol [51]. Maintenance of physiological balance across these interconnected neuroendocrine mediators is essential for mental and behavioral well-being across the lifespan.

b. **Gut Hormones and Neurotransmitters**

Microbial communities may produce precursors to neurotransmitters, drive the synthesis of neurotransmitters via the metabolism of dietary substances, or perform a combination of these functions [21]. For example, the gut microbiota can produce neurotransmitters such as Gamma-Aminobutyric Acid (GABA), dopamine, and serotonin [54]. More than 90% of body’s serotonin, a key neurotransmitter of the brain–gut axis is synthesized in the gut [55]. Bacteria such as *Streptococcus* spp., *Enterococcus* spp., *Escherichia* spp., *L. plantarum*, *Klebsiella pneumonia*, and *Morganella morganii* all have the ability to produce serotonin [55]. Although gut microbes are capable of synthesizing dopamine, this neurotransmitter does not traverse the blood–brain barrier. Consequently, it has been proposed that the gut microbiota modulate brain function through indirect mechanisms, which in turn may influence mood regulation and sleep architecture [56].

c. **Short-Chain Fatty Acids (SCFAs)**

Gut bacteria ferment dietary fibers and produce short-chain fatty acids (SCFAs), essential chemical compounds such as butyrate, acetate, and propionate. SCFAs, when transported through the stomach, may directly influence vagal afferent nerves, which play crucial roles in regulating satiety, stress responses, and mood. In particular, butyrate has emerged as a key neuroactive metabolite that exerts its effects via G protein–coupled receptors, notably GPR41 (free fatty acid receptor 3, FFAR3) and GPR43 (FFAR2), which mediate microbiota–host communication and regulate metabolic, inflammatory, and neuroimmune processes [57]. Furthermore, SCFAs can traverse the blood–brain barrier (BBB) and enter the brain’s bloodstream and cerebrospinal fluid (CSF). This mode of transport may directly affect neurotrophic factor levels, which are crucial for synaptic and neuronal development and differentiation [58]. Recent findings propose that microbial-derived short-chain fatty acids, including butyrate, exert neuroactive effects by enhancing BDNF availability, encouraging neuronal renewal, and contributing to the maintenance of long-term memory [59].

d. **Blood–Brain Barrier (BBB) Integrity**

The BBB is a semi-permeable, highly regulated membrane between the blood and the interstitium of the brain, that plays a critical role in controlling the influx and efflux of biological substances essential for the brain’s metabolic activity as well as neuronal function [60,61]. The BBB appears to develop as soon as cerebral microvasculature begins to form during early embryonic development. According to preclinical research, imbalance in gut microbiota composition is linked to enhanced permeability of the blood–brain barrier (BBB), impacting neurological health. Braniste’s study revealed that germ-free mice, beginning from the intrauterine period, had higher BBB permeability than pathogen-free mice with normal microbiota. This condition persisted after birth and throughout adulthood, accompanied by decreased expression of the tight junction proteins occludin and claudin-5, key regulators of endothelial barrier function [62].

e. **Immune System Modulation**

The interplay between the commensal microbiota and the immune system development and function includes multifold interactions [63]. The gut microbiota plays an important role in the regulation of immune responses by either stimulating the innate immunity through the lymphoid tissue located in the intestine system, or through the interaction between bacterial fragments and receptors placed on the surface of epithelial and immune cells that activate specific systemic and local immune responses [64].

## 4. Development of Sleep: The First 1000 Days

Sleep patterns change rapidly during the first 1000 days for both mother and infant. Pregnancy increases the physiological need for sleep, driven by elevated hormones such as progesterone and human chorionic gonadotropin that promote sleepiness; however, common discomforts and pregnancy-related sleep disorders often fragment rest, creating a paradox of high demand but poor-quality sleep [65]. This sleep disruption perturbs circadian rhythms, leading to alterations in the gut microbiome, specifically reducing beneficial bacteria like *Roseburia* and *Lactobacillus* that produce short-chain fatty acids vital for gut integrity and immune regulation thereby promoting systemic inflammation and metabolic imbalance [66]. Concurrently, sleep loss dysregulates the hypothalamic–pituitary–adrenal axis, elevating cortisol while diminishing melatonin, which is essential for reproductive hormone regulation and neurodevelopmental signaling. These neuroendocrine and microbial changes disrupt metabolic hormones such as ghrelin and leptin, impairing appetite control and insulin sensitivity [67], and collectively increase the risk of adverse pregnancy outcomes. Thus, the intricate interplay between sleep quality, gut microbiota, and neuroendocrine function constitutes a critical triad that influences maternal health and fetal neurodevelopment, underscoring the importance of promoting restorative sleep during pregnancy [68]. In contrast, for children, sleep requirements are highest in infancy, gradually decreasing through toddlerhood and childhood, and continuing to decline until late adolescence, when recommended sleep durations approach those of adults [69]. During early childhood, sleep appears to be both influenced by and a modulator of gut microbial composition and function. Disruptions in early microbial development have been associated with alterations in sleep–wake cycles, circadian rhythm consolidation, and behavioral outcomes. Given that both sleep and gut health are critical during the early days, understanding their interplay may offer new insights into strategies for optimizing neurodevelopmental outcomes through microbiome-targeted interventions.

While fetal eye movements (EMs) can be observed by ultrasonography from 15 weeks gestation [70], infants are born with an immature circadian system [71]. By 10–12 weeks after birth, circadian rhythms begin to emerge, enabling longer nighttime sleep. Total sleep duration decreases from 16–17 h in newborns to 14–15 h by 16 weeks, and 13–14 h by 6 months. As daytime sleep declines, nighttime sleep increases, shifting toward a predominantly nocturnal pattern by the end of the first year [72]. This period of infancy is crucial to establish sleep patterns and through complex interactions involving microbial metabolites (e.g., SCFAs), neuroactive compounds (e.g., serotonin, GABA), modulation of systemic inflammation, and even development of microbial rhythmicity, the gut microbiome exerts significant influence on sleep regulation as well.

A longitudinal study identified a compelling association between gut microbiota diversity and infant sleep patterns. The study investigated the interplay between sleep habits, gut microbiota, and behavioral development in 162 healthy infants at 3, 6, and 12 months of age, with a follow-up behavioral assessment at 24 months. The cohort consisted of term-born, vaginally delivered, and breastfed infants, with no critical confounding health factors. Across the first year of life, *Bifidobacterium* and *Bacteroides* were the predominant bacterial genera. Notably, the gut microbiome underwent a more pronounced compositional shift between 6 and 12 months than between 3 and 6 months. Two primary enterotypes emerged: enterotype A, dominated by *Bifidobacterium*, and enterotype B, dominated by *Bacteroides*. The majority of infants transitioned from enterotype A to B during the second half of the first year. Specifically, higher alpha diversity was linked to fewer and shorter daytime naps, suggesting more mature or “advanced” sleep behavior. This association was the strongest at 3 months of age and diminished thereafter. Additionally, infants with a more mature gut microbiota exhibited increased nighttime activity and awakenings [73]. Associations were observed in another randomized double-blind trial with 161 infants in which the control group received a cow’s milk-based infant formula or a comparable formula with an added prebiotic blend (polydextrose and galactooligosaccharides [PDX/GOS]) from 14–35 to 112 days of age. The study concluded faster consolidation of daytime waking state in infants receiving prebiotics [74]. Correspondingly, the Norwegian Mother, Father and Child Cohort Study (MoBa) reported that children with colic were more likely to get less sleep than recommended (22%) and experienced more frequent awakenings at night (14%) compared to typical children aged 6 months to 5 years [75]. A double-blind, placebo-controlled randomized trial was conducted in Chengdu, China to evaluate the impact of *Bifidobacterium animalis* subsp. *lactis* BB-12^®^ on sleep in breastfed infants with colic. The study enrolled 192 full-term infants younger than 3 months of age who met the ROME III criteria for colic. Following a 1-week run-in, infants were randomly assigned to receive either BB-12 (1 × 10^9^ cfu/day) or placebo for 3 weeks. Sleep outcomes were captured through 24-h structured diaries completed by caregivers. At the end of the intervention, infants supplemented with BB-12 showed a significant improvement in daily sleep duration compared with those receiving placebo, with mean increases of 60.7 ± 104.0 min versus 31.9 ± 102.7 min per day, respectively (*p* < 0.001). In addition to sleep, BB-12 supplementation was associated with reductions in crying/fussing duration and frequency, suggesting that improved sleep may be linked to alleviation of colic symptoms [76]. Another key aspect that links the early infant microbiome with sleep–wake patterns is the gut microbial rhythmicity which emerges early in life and strengthens over a period. A recent study in 162 healthy infants demonstrated that microbial diurnal oscillations track closely with circadian maturation and are associated with the development of the infant’s circadian rhythm. This suggests a link between microbial rhythmicity and host circadian organization during early life. It also stands to reason that an immature or disrupted rhythm may contribute to fragmented sleep by weakening gut–brain timing signals during early development [77]. Together, these studies highlight emerging evidence that gut microbiome modulation can shape infant sleep, with consistent links to nap patterns, sleep consolidation, and night awakenings.

While direct studies that manipulate sleep to examine its effects on the microbiome and subsequent cognitive development in infants and young children remain limited, this area is gaining growing scientific attention. Evidence from studies in older children and adults indicates that healthy sleep is critical for normal brain development and cognitive functioning, supporting key processes such as memory consolidation, attention, executive functioning, and emotional regulation [72,78].

During a typical night, one’s sleep alternates through cycles of Rapid Eye Movement (REM) and Non-Rapid Eye Movement (NREM) sleep about every 90 min [79]. Deprivation of REM sleep has been shown to significantly influence neuronal excitation, a process essential for evaluating potential threats and processing threat-related stimuli. In contrast, NREM sleep deprivation impairs the normal release of certain neurotransmitters, potentially disrupting receptor recovery and sensitivity. The absence of these critical sleep stages ultimately leads to diminished cognitive functioning [80,81]. This concept is relatively straightforward and easy to interpret in individuals with established sleep architecture, such as adults. Drawing clear conclusions in infants is more challenging, as they lack mature circadian rhythms and well-defined REM and NREM sleep cycles. However, a few studies have suggested a potential link between early sleep characteristics and subsequent development of cognitive functions, memory [82] and language skills in infants and young children. For instance, a study was conducted to assess the association of frequency of nighttime awakenings and cognition; it was concluded that frequent nighttime awakenings were associated with poor cognitive function in toddlers. However, a nonlinear association between nighttime awakenings and cognitive performance was found among infants. It is important to note that total sleep duration was not associated with any developmental indices in both infants and toddlers [83]. In one of the first experimental demonstrations of sleep’s role in memory consolidation, 6 and 12-month-old infants who napped for at least 30 min within 4 h of learning showed significantly better declarative memory for novel actions after both 4 and 24-h delays, compared to non-napping peers and control groups. Additionally, memory performance was significantly enhanced in the nap group after the 24-h delay [84]. Similar results have been reported for the impact of sleep on language. An interesting study conducted with twins in Quebec reported that children with language delays at 60 months had less mature sleep consolidation at both 6 and 18 months than children without delays and those with transient early delays [85]. In another longitudinal study infants were assessed at 8 and 14 months, on sleep, cognitive and language skills, and cortisol levels. Findings showed that optimal sleep at 8 months modestly predicted better cognitive and language outcomes at 14 months, highlighting the importance of early sleep patterns for developmental outcomes [86]. Similar results were reported in “The Beijing Longitudinal Study”, where high scores on the Bayley infant development assessment at 6 months predicted less nocturnal awakenings at 1 year of age and insufficient nocturnal sleep at 1 year predicted poor fine motor development at 2 years [87]. The results of the study carried out by Schoch were in line with the other studies. Schoch concluded that both sleep habits and gut microbiota composition were associated with behavioral development, particularly at 3 months. The study also established that sleep patterns were more closely linked to personal-social developmental domains, while gut microbiota composition was more strongly associated with motor development [73] alluding to the impact of sleep on cognition in young children.

Taken together, these studies provide compelling evidence that early-life sleep patterns play a critical role in shaping cognitive, memory, language, and behavioral development in infants and young children. While the architecture of infant sleep is immature and distinct from that of adults, disruptions such as frequent nighttime awakenings or insufficient consolidation have been consistently linked to poorer developmental outcomes. Conversely, timely and sufficient sleep including naps appears to enhance memory consolidation and support language acquisition. Longitudinal findings further emphasize that early sleep quality predicts later cognitive, motor, and social-emotional functioning, underscoring sleep as a foundational process in neurodevelopment. Emerging data also highlights the interplay between sleep, gut microbiota, and developmental trajectories, suggesting that sleep not only reflects but may actively shape broader biological and behavioral systems. Together, these findings position early sleep regulation as a critical window of opportunity for fostering optimal brain development and long-term child wellbeing.

## 5. Nutritional Needs: The First 1000 Days

**Figure 3:**
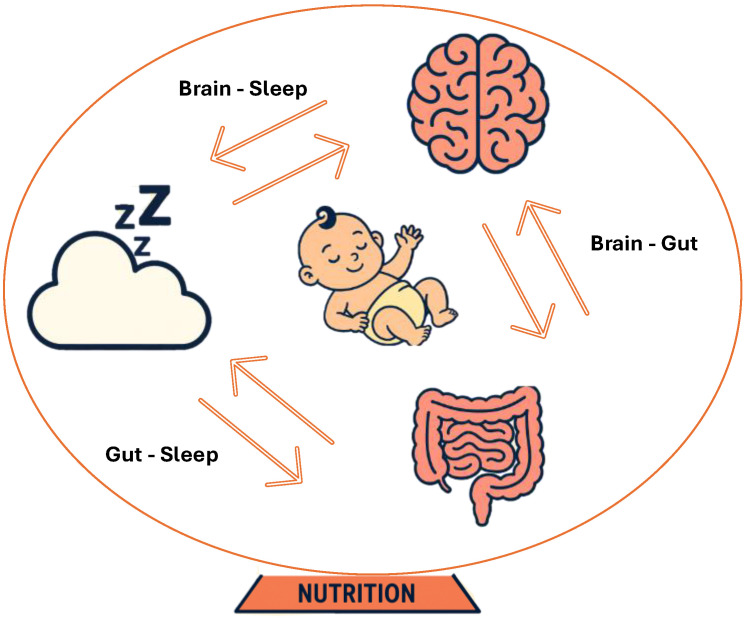
The Brain–Gut–Sleep Axis: Bidirectional Interactions Supported by Nutrition. Nutrition provides a foundational and modifiable influence on the interconnected triad of sleep, gut, and brain development during the first 1000 days of life, thereby shaping neurodevelopmental outcomes later in life.

Nutritional input during the first 1000 days of life is a primary modifiable determinant of the coordinated maturation of the brain, gut, and sleep systems mentioned earlier in this review (Figure 3). Nutrients such as omega-3 fatty acids, choline, folate, iodine, vitamin B12, iron, and vitamin D are essential for optimal neurodevelopment [88,89]. Similarly, nutrients like omega-3 fatty acids, iron and vitamin D can support better sleep, while probiotics and HMOs are known to support a healthy microbiome and potentially lay the foundation for the rest of the lifespan. In this section, the role of key nutrients during the first 1000 days of life is evaluated, along with recommendations provided by expert bodies for some nutrients (Table 1). Whereas existing gaps in guidance and future strategies that should be considered to support healthy maternal–fetal outcomes are summarized later in Table 2.

### 5.1. Omega-3 Fatty Acids

The composition of the human brain includes approximately 60% fat [90], with docosahexaenoic acid (DHA) being the most abundant omega-3 long-chain polyunsaturated fatty acid (PUFA) found in the gray matter, representing about 15% of all fatty acids in the frontal cortex [91]. While endogenous synthesis covers most fatty acids, the body cannot generate omega-3 and omega-6 PUFAs on its own due to missing enzymatic pathways. Thus, essential precursors such as α-linolenic acid (ALA) and linoleic acid (LA) must be supplied through diet or supplementation with DHA and EPA [92]. Maternal dietary intake of DHA is directly proportional to the increase in fetal supply thereby contributing to higher DHA concentrations in cord blood [93]. DHA supplementation during pregnancy and infancy may offer beneficial effects on the development of visual acuity, cognitive functions and other domains of neurodevelopment [94,95], maturity of sleep patterns, spontaneous motor activity and immune phenotypes [96]. In the Kansas University DHA Outcomes Study (KUDOS), mothers were randomized in a double-blind fashion, to receive either 600 mg/d of DHA or a placebo beginning at 14.5 weeks of gestation until delivery. Children from those pregnancies were evaluated for cognitive and behavioral impact from 10 months through 6 years of age. The study reported a dramatic reduction in premature birth and improved visual attention in infancy was identified in the supplemented group, along with a favorable brain response in the Go/No-Go testing at 5.5 years. However, no other pronounced cognitive benefit was seen after controlling for socioeconomic status (SES) [97]. In another triple-blind randomized controlled trial in 150 pregnant women the effects of fish oil supplementation (120 mg DHA and 180 mg EPA) versus placebo were evaluated from the 20th week of pregnancy to 30 days postpartum. The children of mothers who received fish oil supplements showed higher mean scores on all Ages and Stages Questionnaire (ASQ) domains compared to the placebo group, and a statistically significant improvement was specifically observed in the communication domain at 4 months [98]. Although clinical trials on prenatal omega supplementation and subsequent cognitive outcomes in children are limited, clinical studies such as those listed above suggest a positive directional trend supporting its cognitive benefits.

DHA also has an essential role in photoreceptor cells in the retina and is crucial for the growth and survival of photoreceptor cells in the eye. While many studies show that DHA improves vision, the molecular mechanisms are still not fully understood [99]. A double blind, prospective, randomized, and controlled study aimed to test whether maternal DHA supplementation (200 mg/d) during pregnancy improves visual development in healthy term infants, measured through visual evoked potentials (VEPs). One hundred women received either DHA-rich fish oil or a placebo from the 15th week of pregnancy until delivery. While maternal fish oil supplementation did not alter infant DHA status at birth, umbilical cord red blood cell DHA levels regardless of supplementation, were nonetheless associated with maturation of the P100 component of the pattern-reversal VEP [100]. Another study investigated whether DHA supplementation (200 mg/day) in breastfeeding mothers affects infant brain and visual development. Mothers took DHA or a placebo for 4 months postpartum. Supplementation significantly increased DHA levels in maternal milk (to 0.3% of total fatty acids) and infant plasma. The notable benefit was a higher Psychomotor Development Index at 30 months in the DHA group. There were no significant differences between groups in infant visual functions or general neurodevelopment at earlier timepoints [101].

A large observational study of 11,875 pregnant women found a significant association between low maternal seafood consumption (less than 340 g per week) and suboptimal neurocognitive outcomes in their children [102]. Recognizing the importance of dietary sources of DHA in fetal development, the 2020–2025 Dietary Guidelines for Americans (DGA) recommend that pregnant and breastfeeding women consume 8 to 12 ounces of a variety of seafood per week [103]. The guidelines, being largely based on food sources, introduces a key paradox during pregnancy as some shellfish and fish may also be high in mercury and therefore not recommended for pregnant women [104]. Additionally, women following vegan or vegetarian diets often face challenges in meeting DHA requirements through food alone. Therefore, to support future maternal and fetal health, women planning pregnancy should consume at least 250 mg/day of combined DHA and EPA from diet or supplements. Pregnant women should aim at achieving an average intake of at least 200 mg DHA/d till breastfeeding [96]. Low maternal DHA intake or blood levels are associated with increased risk of preterm and early preterm birth and therefore, women at a higher risk should consider supplementing with 600–1000 mg/day of DHA ± EPA [105].

Maternal DHA status directly influences the DHA content of breast milk and should be adequate to meet infant’s requirements [106]. However, as discussed earlier, deficiencies can arise for various reasons, including maternal dietary restrictions such as strict vegan or vegetarian diets. In such cases, direct DHA supplementation for the infant may be necessary to support optimal neurodevelopment. Unlike DHA, arachidonic acid (AA) levels in infants remain largely independent of maternal dietary intake. During the last trimester of pregnancy and the first two postnatal years, a critical window characterized by rapid brain growth, AA and its elongation product, adrenic acid (22:4*n*-6), increase substantially. By early childhood, adrenic acid accounts for nearly half of brain *n*-6 long-chain polyunsaturated fatty acids (PUFAs), and the total *n*-6 PUFA content significantly surpasses that of *n*-3 PUFAs. This highlights the central structural role of AA-derived lipids in brain membrane expansion and maturation, working in concert with adequate DHA levels to ensure proper neural development. Recognizing this essential role, the European Society for Paediatric Gastroenterology Hepatology and Nutrition (ESPGHAN) guidelines emphasize the inclusion of both DHA and AA in infant formulas to support healthy brain development. In a dose-dependent, double-masked, randomized trial [DHA Intake And Measurement Of Neural Development-(DIAMOND Study)] 343 healthy, term, formula-fed infants (1–9 days old) were assigned to one of four formulas varying only in DHA content: 0% (control), 0.32% (17 mg/100 kcal of infant formula), 0.64% (34 mg/100 kcal), or 0.96% DHA (51 mg/100 kcal) all in combination with 0.64% AA. Visual acuity at 12 months was assessed in 244 infants via visual evoked potential. The study concluded that infants who were fed the control formula had significantly poorer visual acuity than those fed any DHA-supplemented formula (*p* < 0.001). However, increasing DHA levels beyond 0.32% did not yield further improvements [107].

A decline in blood levels of omega-3 fatty acids, particularly DHA, is observed between 6 and 12 months of age. This is primarily due to decreasing maternal DHA stores and the introduction of DHA-poor solid foods, which gradually replace human milk as the main source of nutrition. In a randomized clinical trial, breastfed infants at 6 months were assigned to receive either a daily jar (113 g) of baby food enriched with egg yolk containing DHA (115 mg per 100 g) or a control formula. Gravimetric analysis estimated supplemental DHA intake at 83 mg per day in the supplemented group, while the control group received none. Despite many infants continuing to breastfeed for an average of 9 months, red blood cell (RBC) DHA levels dropped significantly in the control group from 3.8 to 3.0 g/100 g total fatty acids between 6 and 12 months. Conversely, infants receiving DHA supplementation showed a 34% increase in RBC DHA levels, from 4.1 to 5.5 g/100 g by 12 months. Visual evoked potential (VEP) acuity improved in supplemented infants, progressing from 0.48 logMAR at 6 months to 0.14 logMAR at 12 months, equating to a 1.5-line advantage on the eye chart over controls. These findings indicate that sustained DHA intake through breast milk and enriched baby foods enhances visual maturation in healthy infants in their first year after birth [108].

Beyond its neurodevelopmental benefits, research indicates that DHA plays a critical role in the regulation of melatonin and serotonin. Evidence from infant studies suggests that maternal DHA supplementation is positively associated with improved sleep quality in offspring [109]. For instance, in a study where maternal plasma phospholipid DHA levels ranging from 1.91% to 4.5% of total fatty acids were categorized as high (>3.0%) or low (≤3.0%), infants of high-DHA mothers demonstrated a lower active sleep (AS) to quiet sleep (QS) ratio, reduced AS, fewer sleep–wake transitions, and greater wakefulness on postnatal day 2. These associations were further supported by consistent correlations of maternal DHA status with infant sleep states. Additionally, a higher maternal *n*-6:*n*-3 fatty acid ratio was inversely associated with QS and positively associated with arousals on day 1, and with increased AS, sleep–wake transition, and AS:QS ratio on day 2, suggesting that maternal DHA status may be an important determinant of early sleep architecture [110]. A randomized, double-blind, placebo-controlled longitudinal study examined the impact of prenatal DHA supplementation via a cereal-based functional food (300 mg DHA, 92 kcal, consumed ~5 days/week) on early neurobehavioral development, specifically infant sleep patterning within the first 48 postnatal hours. Pregnant women between ages 18–35 years (*n* = 27 DHA, *n* = 21 placebo) started the intervention at 24 weeks gestation until delivery (38–40 weeks). Sleep/wake states of infants were recorded on postnatal days 1 and 2 using a pressure-sensitive mattress that tracked respiration and body movements. After controlling for ethnic variation, significantly fewer arousals in quiet sleep on both days (*p* = 0.006 and *p* = 0.011) and in active sleep on day 1 (*p* = 0.012) was reported in the DHA group compared to placebo [111].

In another study of 135 pregnant women, maternal PUFA status specifically the balance between anti-inflammatory DHA and pro-inflammatory AA was linked to gestational length. Women with a lower DHA:AA ratio exhibited shorter gestation and a higher risk of preterm birth, with both inflammatory pathways and maternal sleep quality identified as potential mediators of this relationship. Therefore, concluding better sleep quality in pregnant women with higher DHA:AA ratio [112]. Taken together, these findings highlight a dual role for maternal DHA: beyond supporting maternal sleep quality, its balance with proinflammatory AA appears to influence gestational length through inflammatory pathways. Given that gestational age is a critical determinant of infant microbiome establishment (discussed earlier in the paper), the convergence of these factors underscores a modifiable window through maternal nutrition. Optimizing maternal PUFA status particularly by improving the DHA status may represent a powerful strategy not only to enhance maternal sleep and reduce inflammation, but also to promote healthier gestational outcomes with downstream benefits for infant neurodevelopment and microbiome maturation. Additionally, in the absence of standardized, mandatory guidelines for omega-3 supplementation beyond 6 months of age, there is a compelling need to promote the inclusion of omega-3 rich foods in the complementary diet during this period [113].

### 5.2. Choline

Choline plays a vital physiological role in lipid metabolism and supports the proper functioning of the brain, liver, and muscles [114]. Miniscule amounts of choline are produced by the humans through the hepatic phosphatidylethanolamine *N*-methyltransferase pathway (PEMT) pathway; dietary intake is crucial to meet physiological needs and prevent deficiency [115]. As a precursor of phosphatidylcholine, choline is required for the assembly and secretion of very-low-density lipoproteins (VLDL), thereby enabling hepatic export and systemic transport of lipids, including long-chain polyunsaturated fatty acids critical for fetal development. When choline availability is inadequate, impaired lipid transport can constrain the delivery of these fatty acids to developing tissues, particularly the brain, where they are required for membrane formation and neural maturation. Consequently, choline is required for the development and activity of fetal progenitor cells involved in differentiation, migration, proliferation, and apoptosis [114]. Animal studies suggest that choline supplementation during pregnancy contributes to changes in fetal brain function and is linked to an improvement in postnatal cognitive and behavioral tests. There is also accumulating evidence that choline insufficiency can cause declines in certain learning and memory capacities [116,117]. In addition to its established physiological functions, choline is also a central component of one-carbon metabolism and a regulator of epigenetic processes. Following its conversion to betaine, choline serves as a methyl donor through the betaine–homocysteine methyltransferase pathway, thereby shaping cellular methylation potential by influencing the balance between *S*-adenosylmethionine and *S*-adenosylhomocysteine. This balance is a major determinant of DNA and histone methylation, epigenetic modifications that govern gene expression without changes to the underlying DNA sequence. Increasing body of evidence further suggests that choline availability modulates the epigenetic control of genes involved in circadian regulation [118], which in turn is critical for neurodevelopment. In the United States, women with lower dietary intake of choline (150 mg/day) are at substantially higher risk for having a baby with a NTDs [119], or an orofacial cleft [120] than women with adequate intake. A prospective cohort study on the effect of choline during pregnancy and on child cognition determined that higher gestational choline intake (>328 mg/day) was associated with better child visual memory at age 7 years [121]. In another study, pregnant women in their third trimester were randomly assigned to consume either 480 mg or 930 mg of choline daily until delivery. Infants’ cognitive development was assessed at 4, 7, 10, and 13 months. Infants of mothers who consumed 930 mg choline/day had significantly faster information processing speeds compared to those whose mothers consumed 480 mg/day. Additionally, within the 480 mg group, longer exposure to choline was associated with faster reaction times, indicating a dose–response relationship. These findings suggest that increasing maternal choline intake during late pregnancy can positively impact infant cognitive development [122]. A seven-year follow-up of Caudill’s research revealed that children from the 930 mg/day choline supplementation group outperformed those from the 480 mg/day group on the sustained attention test (SAT). They demonstrated an enhanced ability to maintain accurate signal detection (hits) throughout the 12-min assessment, further supporting the cognitive benefits of higher prenatal choline intake [123].

The adequate intake for choline was first established in 1998; however, better biomarkers for the assessment of choline status are still needed for clinical practice in nutrition [124]. Despite its importance, most women in the U.S. do not meet the recommended intake of choline during pregnancy [125]. This gap is further exacerbated among women following vegetarian or vegan diets, as they rely solely on plant-based sources, which tend to be low in choline. As a result, choline supplementation becomes critical, particularly during pregnancy. Yet, most prenatal vitamins either lack choline entirely or provide insufficient amounts [126]. According to the DGA and American College of Obstetricians and Gynecologists (ACOG), a daily intake of 450 mg of choline is recommended during pregnancy [103] to support maternal and fetal health.

In addition to the above recommendations for pregnancy and fetal outcomes, it is important to further study the effects of choline supplementation in infants directly. In one double-blind RCT conducted in the UK, infants aged 1 to 18 months with suspected cerebral palsy (CP) were enrolled through child development centers. Participants received daily supplementation or placebo for two years. The active supplement was a multi-nutrient formulation containing Choline (10.5 mg in treatment group versus 1.38 mg in control) along with other nutrients known to support neurodevelopment (DHA-1% of estimated total daily fatty acid intake), EPA, AA, uridine monophosphate, cytidine monophosphate, vitamin B12, zinc, and iodine). While the study did not demonstrate a statistically significant neurodevelopmental advantage for the intervention group compared to controls, the treatment group showed cognitive and language improvements of a clinically meaningful magnitude [127]. Efforts such as improved diagnostic tools to detect choline deficiency early on and the implementation of well-designed randomized controlled trials (RCTs) in pregnant populations and in children are essential to generate high-quality evidence, furth guide clinical recommendations.

### 5.3. Folate

Folate is key for optimal brain functioning and plays an important role in mental and emotional health [128]. It is necessary for producing DNA and RNA, particularly during periods of rapid growth such as infancy, adolescence, and pregnancy. Folate also works in tandem with vitamin B12 to produce red blood cells and supports proper iron utilization in the body [129]. Mammals are unable to produce folate on their own, so they must obtain it through supplements to keep healthy levels. A deficiency in folate can result from not eating enough folate-rich foods, problems absorbing folate, or changes in folate metabolism caused by genetic issues or interactions with certain medications. Adequate folate intake is especially crucial during pregnancy, as a woman’s folate needs increase by 5 to 10 times compared to when she is not pregnant. This heightened demand is essential to ensure proper growth and development of both maternal and fetal tissues [130]. While the link between folate deficiency during the prenatal period and an increased risk of NTDs is well documented, the role of folate as it relates to cognition, there is a critical need for long-term, well-designed trials [130,131,132].

A large study (*n* = 3445) examined the relationship between maternal folic acid supplementation during pregnancy and cognitive outcomes in children at age four. The study indicated that children whose mothers began taking folic acid supplements before conception had significantly higher language–social developmental quotients (DQ) compared to those whose mothers did not use supplements at any point during pregnancy. Additionally, children of mothers who initiated folic acid supplementation within the first 12 weeks of gestation showed significantly better outcomes in both cognitive-adaptive and language–social DQ domains compared to non-users. In contrast, no significant association was found between levels of dietary folate intake from preconception to early pregnancy and any cognitive domain when comparing intake levels of 200–400 µg or ≥400 µg to the reference group (<200 µg). Therefore, establishing that early prenatal folic acid supplementation, rather than dietary folate intake alone, is positively linked to improved cognitive development in 4-year-old children [133]. Interestingly, continued supplementation of folic acid beyond the first trimester has shown better cognitive outcomes on tests of word reasoning and cognition. In comparison with a nationally representative sample of British children aged seven years, children whose mothers received folic acid exhibited significantly higher scores on the Wechsler Preschool and Primary Scale of Intelligence-III (WPPSI-III) for verbal IQ (*p*  <  0.001), performance IQ (*p*  =  0.035), general language (*p*  =  0.002), and full scale IQ (*p*  =  0.001). By contrast, the placebo group demonstrated smaller score differences relative to British children in verbal IQ (*p*  =  0.034) and full-scale IQ (*p*  =  0.017) [134]. Findings from other studies [134] underscore the importance of folic acid supplementation during the first trimester and beyond for improved cognitive outcomes in offspring. However, higher doses do not necessarily confer additional benefits; in fact, supplementation exceeding 1000 mcg has been associated with adverse effects in children [135]. These findings highlight the need for appropriate dosing. Accordingly, the DGA [136] recommends a daily intake of 400 micrograms (mcg) of folic acid for all women of childbearing age, including those planning a pregnancy whereas The United States Preventive Services Task Force (USPSTF) recommends that all women who are planning or capable of becoming pregnant take a daily supplement containing 400–800 mcg of folic acid [103].

Dietary supplements may contain either folic acid or 5-methyltetrahydrofolate (5-MTHF), the biologically active form of folate. Folic acid undergoes a multi-step enzymatic conversion to l-methylfolate, with the final step catalyzed by methyltetrahydrofolate reductase (MTHFR). Individuals with certain MTHFR gene polymorphisms exhibit reduced enzymatic activity, which can impair folic acid metabolism and result in suboptimal blood folate levels, high homocysteine concentrations and may have higher requirements for folate and riboflavin [137]. In contrast, 5-MTHF bypasses these enzymatic steps, offering an alternative, particularly for those with compromised MTHFR function. Supporting this, the European Food Safety Authority (EFSA) concluded in 2022 that 5-MTHF is more bioavailable than folic acid at a daily intake of 400 mcg [138]. While mechanistic rationale supports its use, clinical studies directly linking 5-MTHF to NTD prevention is limited and more research is needed.

As explained earlier, human milk provides the exclusive source of folate for infants who are fully breastfed. During lactation, folate transfer into milk is mediated by active transport mechanisms in the mammary epithelium, allowing milk folate concentrations to remain relatively stable even when maternal intake is limited. As a consequence, higher concentrations of 5-methyltetrahydrofolate (5-MTHF) in breast milk have been linked to improved folate status in infants, accompanied by reductions in maternal circulating folate levels [139]. Obeid et al. evaluated the influence of infant methylenetetrahydrofolate reductase (MTHFR C677T) genotype, feeding mode (human milk vs. formula), and the chemical form of folate supplied in formula (L-5-methyltetrahydrofolate or folic acid) on biomarkers of folate metabolism. Outcomes included plasma and red blood cell (RBC) folate, homocysteine, and para-aminobenzoylglutamate (pABG), a catabolite reflecting intracellular folate turnover. Both genotype and folate form were shown to modulate folate biomarkers. Independent of MTHFR genotype, infants receiving formula fortified with L-5-methyltetrahydrofolate experienced a greater increase in RBC folate over the 16-week intervention than those fed an equimolar amount of folic acid. Despite formula folate concentrations being designed to approximate those found in human milk, breastfed infants exhibited lower plasma and RBC folate concentrations, as well as lower plasma pABG, compared with formula-fed infants at the conclusion of the study. The study brings attention to the fact that the form of a nutrient added to the formula, as well as the matrix to which it is added, may well be very important [140].

### 5.4. Iodine

The most severe harm of iodine deficiency is to the developing brain [141], specifically during active myelination of the central nervous system in the perinatal period, and during fetal and early postnatal development. As a result, iodine deficiency is associated with intellectual disability, which in some cases can be severe, and result in impaired cognitive development, intellectual disability, hypothyroidism, goiter, cretinism (neurological damage from fetal hypothyroidism), and other varying degrees of growth and developmental abnormalities [142]. A review of the effects of iodine deficiencies in pregnancy and infancy by Zimmermann [143] reported that iodine supplementation reduces infant mortality in severely iodine deficient populations. In addition, iodine deficiency may impair cognitive and neurological function in the offspring of iodine deficient women. Another meta-analysis also suggested that continued moderate-to-severe iodine deficiency negatively impacted the expected average IQ by about 13.5 points [144]. Another review showed that iodine deficiency at the beginning of gestation is potentially damaging to fetal brain development and is irreversible by mid-gestation unless interventions are initiated promptly to correct the accompanied maternal hypothyroxinemia [145]. The iodine intake of a pregnant woman needed to increase by about 50% to produce enough thyroid hormones to meet both her own and her baby’s requirements [146]. Studies in Papua New Guinea and in Andean regions, where goiter and cretinism are common, showed pregnant women were unable to increase their circulating thyroxine at the onset of pregnancy, again laying focus on early interventions to avoid the lowered intelligence quotient suffered by the inhabitants of the areas [145].

Another observational study followed 1040 mother-child pairs of the Avon Longitudinal Study of Parents and Children (ALSPAC) by measuring iodine concentration in stored spot-urine samples from the first trimester of pregnancy to investigate the relationship of those values to the child’s cognitive performance at 8–9 years of age. Verbal, performance and total IQ was assessed at 8 years of age, using an abbreviated form of the Weschler Intelligence Scale for Children (WISC-III), with reading speed, accuracy, and comprehension being assessed at age 9 by trained psychologists, using the Neale Analysis of Reading Ability (NARA II). Results of this study showed a higher percentage of children born to women with a lower iodine status during pregnancy, including those classified as having a mild-to-moderate deficiency, had suboptimal cognitive outcomes; meanwhile, a significant trend towards a lower risk of suboptimal classification with increasing maternal iodine status was also observed [147].

Ensuring adequate iodine intake before pregnancy is vital, with evidence showing that this supports healthier thyroid hormone status throughout gestation. Research from Italy revealed that women who had been using iodized salt consistently for two years before conception showed improved thyroid hormone levels, increased urinary iodine concentration (115 µg/L versus 63 µg/L), and fewer thyroid disorders (6.4% compared to 36.8%) than women who started iodized salt intake at pregnancy onset [148]. Growing evidence shows that even mild gestational iodine deficiency (GID) harms children’s neurocognitive development. A longitudinal analysis of the Gestational Iodine Cohort (*n* = 266) evaluated long-term educational outcomes in children whose prenatal development occurred during a period of mild iodine insufficiency, despite subsequent exposure to an iodine-sufficient environment. The study investigated whether earlier associations between mild gestational iodine deficiency (GID) and reduced literacy performance observed at 9 years remained evident during adolescence. Offspring were categorized based on maternal gestational urinary iodine concentrations (UICs) above or below 150 µg/L. Literacy and numeracy outcomes were derived from Australian National Assessment Program Literacy and Numeracy (NAPLAN) scores. The findings indicated that children of mothers with UICs < 150 µg/L experienced enduring reductions in spelling performance, with a 10% deficit evident in Year 3 (−41.4 points; 95% CI −65.1 to −17.6; *p* = 0.001) and a 5.6% deficit persisting through Year 9 (−31.6 points; 95% CI −57.0 to −6.2; *p* = 0.015), compared with peers whose mothers had sufficient iodine status during pregnancy [144]. A Norwegian study concluded that there is an association between having a low UIC (µg/L) in pregnancy (lower than ~100 µg/L) and poorer skills in language domains (receptive and expressive) in infancy and toddlerhood [149]. In a clinical study conducted in 2012, the association between maternal UIC during early pregnancy and executive functioning in children at 4 years of age was examined. In addition, the modification of this association by maternal diet and thyroid function was investigated. During pregnancy, UIC and thyroid hormone concentrations in 1156 women were measured. Executive function was evaluated in 692 children using the Behavior Rating Inventory of Executive Function, while dietary intake data were collected from 500 mothers of Dutch origin through a food frequency questionnaire. Associations were examined using regression-based analyses. Results indicated that offspring of mothers with lower urinary iodine concentrations (UIC) during pregnancy exhibited greater difficulties in executive functioning, particularly in domains related to inhibitory control [β = 0.05; 95% CI: 0.01–0.10; *p* = 0.03] and working memory [β = 0.07; 95% CI: 0.02–0.12; *p* = 0.003]. These findings suggest that insufficient maternal iodine status during gestation is linked to poorer executive function outcomes in children [150]. The Japan Environment and Children’s Study also highlighted a similar trend where the infants whose mothers fell into the lowest and second-lowest quintiles of iodine intake (41–123 µg/Day) had a higher likelihood of delayed problem-solving skills at 1 year of age, whereas those in the highest iodine intake quintile (≥277 µg/Day) showed a reduced risk. Whereas children in their third year, presented signs of delay in communication, fine motor, problem-solving, and personal–social domains from the lowest and second quintile iodine intake group compared with the fourth quintile iodine intake group, while the risk of delay for fine motor and problem solving domains was decreased in the highest quintile iodine intake group [151]. A supplementation trial examined early neurodevelopmental outcomes in infants aged 3 to 18 months whose mothers’ received iodine during pregnancy. Women were divided into two groups: 133 mothers who were given 300 µg of potassium iodide during the first trimester and 61 mothers who did not receive iodine supplementation. Infant neurodevelopment was assessed using the Bayley Scales of Infant Development, alongside measurements of maternal and infant thyroid-related biomarkers, including thyroid-stimulating hormone (TSH), free triiodothyronine (T3), free thyroxine (T4), and urinary iodine concentration (UIC). Infants born to mothers who received iodine supplementation demonstrated more favorable neurodevelopmental outcomes, with significantly higher scores on the Psychomotor Development Index (*p* = 0.02) and improved performance on the Behavior Rating Scale compared with infants whose mothers were not supplemented. These findings indicate that iodine supplementation during pregnancy positively influences early childhood neurodevelopment [152]. Several other studies and review articles help establish a positive association between adequate iodine maternal levels and cognitive outcomes in offsprings [143,153,154].

There is a broad consensus on the importance of adequate iodine intake for improving maternal and fetal outcomes. Women who are pregnant or planning to conceive should consume 220 mcg/day of iodine, starting from the preconception period and continuing throughout pregnancy [136]. Childbearing women should be encouraged to use iodized salt and choose prenatal supplements that contain iodine. Breast milk is the primary source of iodine for infants in this category. Maternal iodine intake directly influences the iodine concentration in breast milk. Consequently, it is recommended that breastfeeding mothers maintain the same iodine intake as during pregnancy to ensure that the infant receives an adequate iodine supply throughout the first six months of life.

### 5.5. Vitamin B12

Vitamin B12 is vital for humans. Adequate intake reduces the incidence of neurological diseases, birth defects and chronic disorders, and is vital for maintaining the brain health [155]. Vitamin B12 is a critical nutrient, alongside folate, riboflavin and vitamin B6, as these interact as cofactors within the one-carbon metabolism, which is a network of interrelated cellular pathways that influence offspring development through roles in biosynthesis of DNA, amino acids, and other molecules, epigenetic modification, and the remodeling of placental function [156,157]. Pregnancy may add another layer of complexity leading to a steady decline of vitamin B12 due to increased fetal demand, hemodilution, and changes in vitamin B12 binding proteins [158]. Research demonstrates that maternal vitamin B12 stores have been linked to positive birth outcomes. In the ECLIPSES prospective cohort study of 434 mother–infant pairs from northern Spain, researchers investigated the association between maternal vitamin B12 status during pregnancy and infant neurodevelopment at 40 days postpartum. Maternal vitamin B12 levels were measured in the first and third trimesters, and infants were assessed using the Bayley Scales of Infant Development-III (BSID-III). After adjusting for relevant covariates, infants whose mothers had moderate first-trimester B12 levels (312–408 pg/mL; tertile 2) showed significantly better motor, gross motor, language, and cognitive outcomes compared to those in the lowest tertile (<312 pg/mL). The likelihood of scoring above the 75th percentile in motor and receptive language domains was also higher in this group. These findings underscore the importance of adequate maternal vitamin B12 status early in pregnancy for optimal early neurodevelopment [159]. Long-term developmental outcomes were examined in offspring participating in the Avon Longitudinal Study of Parents and Children. Maternal vitamin B12 intake during pregnancy was stratified, and children born to women in the lowest 10% of intake consistently demonstrated poorer performance across several domains of language and cognitive development. Deficits were evident in early language acquisition, including reduced vocabulary at 2 years and limited phrase construction by 38 months. As children aged, additional challenges emerged, such as diminished speech clarity at 6 years and weaker numeracy skills during later primary school years (ages 8–11). By adolescence, these children also achieved lower scores on standardized national mathematics assessments at 13 years of age. Together, these findings provide evidence that inadequate maternal vitamin B12 intake during pregnancy is associated with persistent adverse effects on offspring cognitive and academic development [160].

A randomized, placebo-controlled clinical trial examined the effects of maternal vitamin B12 supplementation (50 µg/day from <14 weeks gestation to 6 weeks postpartum) on infant cognitive development at 9 months. The study concluded that higher maternal total homocysteine levels in the second and third trimesters were significantly associated with expressive language and gross motor domains. However, the study did not show significant differences in cognitive scores, language, or motor scores between the B12 and placebo groups [161]. In another study focusing on the “preconception” period, the Pune Rural Intervention in Young Adolescents (PRIYA) trial (*n* = 557), adolescent participants received daily supplementation of vitamin B12 (2 µg), with or without multiple micronutrients (MMN), or placebo from preconception to delivery. All groups were provided with standard iron and folic acid. Neurodevelopment of offspring was assessed at 24–42 months using the BSID-III. Offspring of mothers in the B12-only group showed significantly better cognitive (*p* = 0.044) and language (*p* = 0.020) outcomes compared to the placebo group, after adjusting for maternal baseline B12 levels. However, no such benefit was observed in the B12 + MMN group [162].

Women who are of childbearing age or are pregnant are at a higher risk of vitamin B12 deficiency especially if they follow dietary patterns such as vegan or vegetarian. The ACOG and the DGA recommends that pregnant women consume 2.6 mcg of vitamin B12 daily. Vitamin B12 is available in several supplemental forms, including cyanocobalamin, methylcobalamin (MeCbl), adenosylcobalamin (AdCbl), and hydroxocobalamin (OHCbl). Cyanocobalamin is commonly used synthetic form due to its stability and cost-effectiveness, although it requires conversion in the body to active forms. Methylcobalamin and adenosylcobalamin are bioactive forms that do not require this conversion. Data comparing the different forms of B12 concluded that supplementing with any of the nature bioidentical forms of vitamin B12 (MeCbl, OHCbl, and/or AdCbl) is preferred instead of the use of CNCbl [163,164]; however, more research on the forms is warranted. A recent Cochrane review, which included randomized controlled trials with vitamin B12 supplementation dosages ranging from 5 mcg/day to 250 mcg [165], did not report any serious adverse events.

In infants, however, vitamin B12 deficiency is largely dependent on maternal stores. As stated earlier, vitamin B12 supplementation is an effective strategy to improve the maternal blood levels of vitamin B12 that could translate to better vitamin B12 levels in the breastmilk [166].

### 5.6. Iron

Iron is an essential mineral for humans, mostly found in complex structures attached to proteins. It is necessary for important biochemical processes like making hemoglobin and myoglobin (which help carry oxygen), creating heme enzymes, and supporting other iron-based enzymes that handle electron transfer and redox reactions. In the human body, around 60% of iron is in the hemoglobin of red blood cells, about 15% is stored in muscle myoglobin, and the remaining 25% is kept in reserve. Iron concentrations must be tightly regulated because excessive amounts can lead to tissue damage, while deficiencies can lead to numerous disorders [167]. Pregnancy significantly increases the risk of negative iron balance due to substantially elevated iron demands compared to the nonpregnant state. This heightened requirement arises from two primary factors: the rapid growth of the fetoplacental unit, and the maternal expansion of blood volume. Approximately 1 g of iron must be accumulated throughout pregnancy. Of this, around 360 mg is transferred to the fetus. Evidence indicates that inadequate iron availability during critical developmental windows, particularly within the first 1000 days of life, can have lasting consequences for neurodevelopment, affecting cognitive performance, motor skills, and behavioral outcomes [168,169]. However, emerging data suggests that iron requirements are not uniform across individuals, raising important questions about whether standardized supplementation strategies are appropriate or whether a more individualized approach is warranted for nutrients such as iron.

Support for a tailored strategy comes from research examining prenatal iron supplementation adjusted according to maternal iron status. In this study, maternal iron stores at the beginning of pregnancy were used to guide supplementation levels, and child neurodevelopment was evaluated using standardized cognitive and neuropsychological assessments. Among women with depleted iron reserves (serum ferritin < 15 µg/L), higher-dose iron supplementation (80 mg/day) was associated with improved cognitive outcomes across a wide range of domains. In contrast, when this same dosage was provided to women with high baseline iron stores (>65 µg/L), children exhibited poorer performance in several cognitive areas, including language-related skills, executive function, and processing efficiency. Notably, within this iron-replete group, a lower supplementation dose (20 mg/day) was associated with more favorable outcomes, including better working memory, overall intellectual functioning, verbal fluency, and emotion recognition [170]. Beyond neurodevelopmental considerations, iron supplementation may also influence gut microbial ecology. A substantial proportion of dietary non-heme iron escapes absorption in the small intestine and reaches the colon, where it becomes available to resident microorganisms. Many pathogenic bacteria, including *Escherichia coli*, *Salmonella*, and *Shigella*, possess strong iron-scavenging capabilities and may preferentially proliferate in iron-rich environments. In contrast, commensal taxa such as *Bifidobacterium* and *Lactobacillus* have relatively low iron requirements and play a critical role in maintaining colonization resistance and intestinal barrier function. Elevated iron availability may also promote the growth of non-bacterial pathogens, such as the malaria parasite *Plasmodium falciparum* [171]. More specifically, prenatal iron supplementation, in anemic subjects, has been linked to improved cognitive performance in children particularly during early developmental stages, including infancy, toddlerhood, and early school years [83]. Collectively, these observations reinforce the need for precision-based prenatal iron supplementation strategies that consider maternal iron status, not only to support optimal neurodevelopment in offspring but also to minimize unintended effects on microbial balance and pathogen susceptibility.

Currently, both the ACOG and DGA recommend a higher daily intake of iron (27 mg/day) for pregnant women, compared to 18 mg/day for non-pregnant women. These recommendations are based on the needs of healthy pregnant individuals, yet they may not fully account for women with conditions such as [83], obesity [172] and gestational diabetes [173], where iron metabolism can differ significantly.

When maternal iron stores are insufficient, infants are often born with low iron reserves, placing them at risk of anemia and its potential developmental consequences. Encouragingly, timely iron supplementation in these infants can help reverse some of the adverse neurodevelopmental effects associated with early iron deficiency [174]. One proposed mechanism is that iron influences early brain development by supporting attentional processes, which undergo rapid maturation and are particularly sensitive to nutritional status during infancy. Approximately 80% of a term infant’s total body iron is accreted during the third trimester of pregnancy. Preterm infants, therefore, miss this critical period of rapid iron accumulation and are often born with significantly reduced iron stores. In addition, certain maternal conditions such as anemia, hypertension with associated intrauterine growth restriction (IUGR), or diabetes during pregnancy can further compromise fetal iron endowment in both term and preterm infants [175]. Consequently, preventing iron deficiency during the prenatal and early postnatal periods may be more beneficial for neurocognitive outcomes than attempting to reverse deficiencies after they occur [176]. A total of 285 infants were randomly assigned to receive daily iron doses of 0, 1, or 2 mg/kg beginning at six weeks of age and continuing until six months. Neurodevelopmental follow-up was conducted at 3.5 years of age and included standardized cognitive testing using the Wechsler Preschool and Primary Scale of Intelligence, along with caregiver-reported assessments of behavioral functioning using the Child Behavior Checklist. Outcomes were compared with those of 95 children born at normal birth weight. The results demonstrated that low-birth-weight children who received iron supplementation during infancy exhibited a lower prevalence of behavioral difficulties compared with unsupplemented peers, suggesting a protective effect of early iron exposure on later behavioral outcomes [177]. McCann and Ames’s 2007 [178] review provided a thorough evaluation of the evidence regarding whether iron deficiency during developmental periods is causally linked to cognitive or behavioral impairments. Drawing on both human and animal studies, the authors concluded that while some evidence supports a causal relationship, particularly in animal studies, more research is needed to solidify the connection in humans, especially regarding cognitive function [178].

As discussed earlier, maternal iron stores are a critical determinant of cognitive development during infancy. With sleep increasingly recognized as a key component of neurodevelopmental health, current evidence highlights a potential link between iron status and sleep outcomes, extending to both mothers and infants. A study among pregnant women attending public health facilities in Bahir Dar City, Ethiopia, assessed sleep quality using the Pittsburgh Sleep Quality Index (PSQI) and reported a significant association between low hemoglobin levels (AOR  =  1.92) and poor sleep quality. Other contributing factors included older maternal age (AOR  =  3.62), third trimester of pregnancy (AOR  =  2.83), multigravidity (AOR  =  2.55), and coffee consumption (AOR  =  2.19) [179]. These findings are consistent with several other studies demonstrating a correlation between low hemoglobin or poor iron status and impaired sleep outcomes in pregnant [180] and reproductive-age women [181].

Iron deficiency anemia (IDA) during pregnancy may also compromise the iron reserves of the fetus, with potential implications for postnatal sleep [182]. Evidence from two randomized, placebo-controlled trials conducted in Pemba Island, Zanzibar, and Nepal showed that infants supplemented daily with iron–folic acid, with or without zinc for 12 months exhibited longer nighttime and total sleep duration compared to controls. Zinc supplementation showed similar benefits [183]. In contrast, a separate longitudinal study investigating the long-term effects of IDA in infancy found that, despite subsequent iron supplementation, affected infants displayed persistent alterations in neural regulatory mechanisms governing sleep–wake cycles and spontaneous motor activity during the preschool years [184].

Taking into account that iron is the world’s most common single-nutrient deficiency, it is important to minimize IDA and iron deficiency among infants and toddlers. As such, the current recommendations advise that both exclusively breastfed and formula fed term infants receive iron supplementation at a dose of 1 mg/kg/day, beginning at 4 months of age and continuing until iron-rich complementary foods are adequately introduced into the diet [175].

### 5.7. Vitamin D

Vitamin D, traditionally known for regulating calcium balance, exerts pleiotropic effects across multiple tissues, including the brain and gastrointestinal tract [185]. Emerging evidence suggests that vitamin D supports cognitive function through neuroprotective mechanisms involving modulation of oxidative stress, calcium signaling, and inflammatory pathways [186], partly by enhancing antioxidant defenses such as glutathione and superoxide dismutase. Beyond skeletal health, vitamin D–vitamin D receptor (VDR) signaling also regulates gut microbiome composition. Animal studies demonstrate that VDR activity maintains intestinal homeostasis by supporting butyrate-producing bacteria, while the immunomodulatory properties of vitamin D indicate a role in early-life microbial colonization [187]. In a clinical study of one-month-old infants, maternal vitamin D supplementation and higher maternal plasma 25-hydroxyvitamin D concentrations were inversely associated with *Bifidobacterium* spp. abundance and positively associated with *Bacteroides fragilis* group counts. Additionally, lower *Clostridioides difficile* levels were observed in vitamin D-supplemented, breastfed infants, particularly among families characterized by alternative lifestyle practices [188]. In pregnant women, Vitamin D readily crosses the placenta, making the mother the exclusive source of this nutrient for the developing fetus. Studies have demonstrated that when maternal serum 25-hydroxyvitamin D [25(OH)D] levels fall below 50 nmol/L, a threshold indicative of deficiency, the fetus is also likely to be deficient [189,190]. Given the crucial role of vitamin D in early development, such deficiencies during this crucial window of life may have lasting implications on the child’s cognitive trajectory. In a study that investigated the association between total circulating [25(OH)D concentrations and neurodevelopmental outcomes in children aged 3 to 5 years, pregnant participants were randomized to receive daily vitamin D_3_ supplementation at doses of 400 IU, 2000 IU, or 4000 IU. Offspring underwent neurodevelopmental evaluation using the Brigance Screen at ages 3–5 years, and 25(OH)D concentrations were assessed at birth and at the time of testing. The analysis examined the relationship between Brigance scores and both 25(OH)D levels and vitamin D binding protein (VDBP) genotype. Findings indicated that higher 25(OH)D concentrations at the time of assessment were significantly associated with better overall neurodevelopmental performance, as reflected by the Brigance quotient (B = 0.208, *p* = 0.049). Sub-score analysis revealed that children of mothers supplemented with 2000 IU/day of vitamin D_3_ scored significantly higher on the language component of the Brigance assessment compared to those in the standard dose group (B = 4.667, *p* = 0.044) [191]. A study on vitamin D’s metabolic role in early neurodevelopment enrolled pregnant women (10–18 weeks gestation) who received either 4000 IU/day of vitamin D_3_ or a placebo, along with a standard prenatal multivitamin with 400 IU vitamin D_3_. Supplementation continued until delivery, and offspring were followed up quarterly by questionnaires and annually with in-person assessments. Given that language and communication delays are hallmark features of various neurodevelopmental disorders, particularly autism spectrum disorder (ASD), early childhood communication skills were used as a proxy for neurodevelopmental delay. The findings suggested a potential neuroprotective role of prenatal vitamin D supplementation in reducing the risk of ASD and other cognitive impairments [192]. In human studies, maternal vitamin D deficiency has been associated with subtle cognitive and psychological impairments in offspring. Current evidence from animal and human studies highlights promising avenues for further research to clarify causality, define critical windows, and identify opportunities for intervention [189], particularly given the strong correlation between maternal and neonatal vitamin D status [193].

A double-blind randomized clinical trial, the Vitamin D Intervention in Infants (VIDI) study, was conducted at a single center in Helsinki, Finland, to examine the long-term effects of early vitamin D3 supplementation. Infants were randomized to receive either a standard dose (400 IU) or a high dose (1200 IU) of oral vitamin D3 daily from 2 weeks to 24 months of age. Follow-up assessments at ages 6 to 8 years revealed that those who received the higher dose of vitamin D3 had a reduced risk of developing internalizing psychiatric symptoms compared to the standard-dose group [194]. Given the growing evidence supporting the role of vitamin D in various aspects of development, including cognitive and behavioral domains, the American Academy of Pediatrics (AAP) recommends that all infants whether breastfed or formula-fed receive 400 IU of vitamin D daily from birth. This supplementation should continue until the infant’s daily intake includes at least 1 L of vitamin D fortified formula or milk [195].

The role of vitamin D in affecting sleep patterns is receiving growing attention with low serum levels linked to poor sleep outcomes [196]. However, evidence during pregnancy remains limited. In a study of 890 pregnant women from the prospective Growing Up in Singapore Towards Healthy Outcomes (GUSTO) cohort, plasma 25-hydroxyvitamin D (25OHD) concentrations were measured at 26–28 weeks’ gestation, alongside assessment of sleep quality using the Pi PSQI and 24-h dietary recall. Plasma 25OHD status was categorized as sufficient (>75 nmol/L), insufficient (50–75 nmol/L), or deficient (<50 nmol/L), and poor sleep quality was defined as a global PSQI score > 5. The study concluded that after adjusting for confounders, women with 25OHD deficiency had significantly higher odds of poor sleep quality (OR 3.49; 95% CI 1.84–6.63) [197]. A systematic review indicated that vitamin D deficiency is directly associated with poor sleep quality and postpartum depression during and after pregnancy [198]. Another study found that vitamin D and folate deficiencies were linked to restless leg syndrome (RLS) in pregnant women, potentially contributing to persistent moderate-to-severe postpartum RLS symptoms and poor sleep outcomes [199].

While there is clear mechanistic data suggesting a role for vitamin D in sleep regulation, there is a notable lack of intervention studies investigating whether vitamin D supplementation improves sleep outcomes during pregnancy in mothers and in infants whose mothers may have received vitamin D supplementation during pregnancy. This is an area that merits further clinical evaluation.

### 5.8. Prebiotics and Probiotics

Prebiotics are indigestible compounds that promote the growth of beneficial gut bacteria, while probiotics are live microorganisms that support host health when present in adequate amounts [200]. Within the first 1000 days, *Bifidobacterium* and *Lactobacillus* are regarded as the two most important microbial species due to their unique abilities to process HMOs, modulate the immune system, and produce beneficial metabolites [201]. As early colonizers of the infant gut, they have been closely associated with favorable fetal outcomes (discussed earlier in the paper). A 2024 JAMA Pediatrics clinical trial (PRIMAL study) showed that administering *Bifidobacterium infantis*, *Bifdidobacterium lactis* BB-12, and *Lactobacillus acidophilus* La-5 at a dose of 1.5  ×  10^9^ colony-forming units (CFU) of each strain, to preterm infants helped shift their microbiome toward a healthier, eubiotic state typical of full-term infants [202]. Another double-blind, placebo-controlled study tested whether probiotics could offset the effects of antibiotics or cesarean birth on infant microbiota. Pregnant women and their infants received either a multispecies probiotic, consisting of *Bifidobacterium breve* Bb99 (2  ×  10^8^ cfu) *Propionibacterium freundenreichii* subsp. *shermanii* JS (2  ×  10^9^ cfu), *Lactobacillus rhamnosus* Lc705 (5  ×  10^9^ cfu) and *Lactobacillus rhamnosus* GG (5  ×  10^9^ cfu) or a placebo. The probiotic significantly affected microbiota composition, with breastfed infants showing increased Bifidobacteria and decreased *Proteobacteria* and *Clostridia*. In the placebo group, both birth mode and antibiotic use disrupted microbiota, reducing *Bifidobacterium*. The study concluded that probiotics eliminated or lessened these negative effects [31]. Beyond the general benefits of probiotic supplementation in supporting a balanced infant gut microbiome, evidence also suggests potential advantages for overall quality of life, including reduced symptoms of colic [76,203,204], supporting immune function, reduction in food allergy risk [205] and neurocognitive benefits. In an open-label study conducted in rural Tanzania, researchers investigated the impact of daily consumption of micronutrient-supplemented probiotic yogurt on maternal and neonatal microbiota. The study enrolled 56 pregnant women during their last two trimesters and continued through one month postpartum. Of these, 26 participants received the yogurt intervention, while 30 served as untreated controls. The study concluded that infants born to mothers in the yogurt group demonstrated a significant shift in gut microbial composition, characterized by an increased relative abundance of *Bifidobacterium* and a reduction in pathogenic bacteria, *Enterobacteriaceae* [206]. The Mothers and Infants Linked for Healthy Growth (MILk) Study enrolled mother–infant pairs who were exclusively breastfeeding to explore potential links between maternal factors and early neurodevelopment. Neural activity in infants was measured using electroencephalography (EEG), while recognition memory was examined through event-related potential (ERP) paradigms that contrasted responses to familiar and unfamiliar auditory stimuli at 1 month of age and visual stimuli at 6 months. Findings indicated that exposure to maternal probiotics was associated with measurable differences in infant neural processing at 6 months. In particular, infants who received indirect probiotic exposure through breast milk during the postnatal period (from 1 to 6 months) exhibited a more pronounced separation in ERP responses to novel versus familiar visual cues within the late slow-wave component, suggesting more efficient updating of memory representations [207]. Similarly, greater frequency of breast milk feedings as early as 1 month have been shown to contribute to infant cognitive development, an effect that was attributed to greater exposure to HMO 2′FL [36]. Several studies have shown that HMOs comprising six monosaccharides glucose (Glc), galactose (Gal), *N*-acetylglucosamine (GlcNAc), *N*-acetyl-D-galactosamine (GalNAc), fucose (Fuc) and sialic acid (Sia) crosslinked with *N*-acetylneuraminic acid (Neu5Ac) serve as a vital nutrient source bacteria in the gut and for brain development. In addition, they act as decoy receptors stopping pathogens from attaching to host mucosal surfaces with the ultimate effect of supporting immune response [208]. It is clear from the studies presented above that there are potential benefits of probiotic use, both alone and together with HMOs during the first 1000 days for infant health and development. This is an intervention opportunity that may serve to improve feeding practices and nutrition to optimize infant development including cognitive abilities and learning potential.

## 6. Conclusions

Taken together, the evidence in the literature suggests that prioritizing nutrient adequacy through the conception-to-infancy developmental continuum can result in improved lifetime outcomes across gut, brain and sleep functions. By treating nutrition as a shared resource for brain, gut, and sleep development, there are significant gains to be made in improving child health that may even extend into adulthood. While associations between specific nutrients (e.g., DHA, Choline and others) and early-life outcomes are consistent, here we have attempted to also highlight potential gaps such as the absence of standardized recommendations for nutrients like omega-3 beyond six months of age, the lack of reliable biomarkers for detecting deficiencies early such as for choline & vitamin B12, and dearth of meaningful clinical data to deepen our understanding of the various forms of nutrients. Integrative approaches that align dietary assessments, standardized dosing and age-appropriate interventions with longitudinal neurocognitive, microbiome, and sleep endpoints are therefore essential. Such an approach will allow for greater precision in dietary strategies and improve our understanding of the complex interplay between nutrition, gut microbiome, sleep, and neurodevelopment during this critical window of life.

## Figures and Tables

**Table 1:** U.S. Guidelines on Nutrient Intake in the First 1000 Days.

Nutrients	Recommendations */Day				
	Pregnancy & Lactation		Year 1		Year 2
	270 Days		365 Days		365 Days
	Pregnant Women	Breastfeeding Women	Breastfeeding Infants	Complementary Feeding	
**Omega-3 Fatty acids**			0–6 months	6–12 months	
**DHA + EPA (mg)**	250–375 ^	250–375 ^			
**DHA alone (mg)**	200–300	200–300	Not Established		
**Choline (mg)**	450 **^b,c^**–550 **^a^**	550 **^a,b^**	150 **^a^**	150 **^a,b^**	200 **^a,b^**
**Folate (mcg DFE)**	600 **^a,b,c^**	500 **^b^**–600 **^a^**	80 **^a^**	80 **^a,b^**	150 **^a,b^**
**Iodine (mcg)**	220 **^b,c^**–290 **^a^**	290 **^a,b^**	130 **^a^**	130 **^a^**	90 **^a^**
**Vitamin B12 (mcg)**	2.6 **^b,c^**–2.8 **^a^**	2.8 **^a,b^**	0.5 **^a^**	0.5 **^a,b^**	0.9 **^a,b^**
**Iron (mg)**	27 ^**a,b,c**^	9 **^b^**–27 **^a^**	11 **^a^**	11 **^a,b^**	7 **^a,b^**
**Vitamin D (mcg)**	15 **^a,b,c^**	15 **^a,b^**	10 **^a,b^**	10 **^a,b^**	15 **^a,b^**

a: Food and Drug Administration (21 CFR Part 101). b: Dietary Guidelines for Americans, 2020–2025. c: American College of Obstetricians and Gynecologists. ^: Recommendation is made in terms of seafood (8 to 12 ounces (2–3 servings) of a variety of low-mercury seafood per week). * Recommendations are based on guidelines by bodies such as Food and Drug Administration (21 CFR Part 101), DGA, ACOG for healthy population only. Pregnant women with co-morbidities such as gestational diabetes, risk of preeclampsia, obesity and preterm infants and/or with health conditions are excluded.

**Table 2:** Existing Gaps and Future Strategies for Supporting Outcomes in the First 1000 Days.

Key Nutrients	Role	Evidence Strength	Research Gaps
Neurodevelopmental Outcomes			
Omega-3 Fatty acids	Neuronal membrane structure; synaptic maturation	Strong evidence from RCTs and systematic reviews; heterogeneity exists for a dose dependent outcome	Absence of standardized, mandatory guidelines for omega-3 supplementation beyond 6 months of age.
Choline	Neural stem cell proliferation; apoptosis regulation; brain and spinal cord development; NTD and memory risk modulation; Choline (as phosphatidylcholine) is essential for VLDL assembly and hepatic lipid export	Moderate evidence from observational studies; limited RCTs in humans.	Need for better biomarkers for assessment of choline status for clinical practice in nutrition and limited number of randomized controlled trials (RCTs) specifically investigating the effects of choline supplementation alone on infant neurodevelopment.
Folate	Cell proliferation; DNA/RNA synthesis; neurotransmitter formation; nervous system development; NTD prevention	Strong evidence for folic acid in NTD prevention; limited evidence on other folate forms.	Absence of clinical studies directly linking different forms of folic acid such as 5-MTHF to NTD prevention
Iodine	Thyroid hormone synthesis; brain and central nervous system growth and maturation	Strong evidence from population-level interventions; deficiency clearly linked to deficits.	Limited data on optimal supplementation levels in iodine-sufficient populations.
Vitamin B12	DNA synthesis; myelination; synaptogenesis; neural connectivity	Moderate evidence: deficiency linked to adverse outcomes, but supplementation trials inconsistent.	Lack of long-term, well-designed studies to determine the efficacy and safety of vitamin B12 supplementation during pregnancy for both maternal health and fetal developmental outcomes—particularly on the various forms of B12.
Iron	Myelination; monoamine neurotransmitter synthesis; energy metabolism in the developing brain	Strong evidence that deficiency impairs cognition; supplementation helps but effect size varies.	Limited evidence on timing/dosing of supplementation in non-deficient populations.
Vitamin D	Neuroprotection; oxidative stress modulation; calcium homeostasis; inflammation regulation	Emerging evidence for cognition; observational links strong.	Lack of long-term, dose dependent clinical trials focusing on neurodevelopmental outcomes.
Probiotics	Gut–brain axis modulation; cognitive support, particularly in preterm infants	Strong emerging evidence	Long-term trials in healthy infants
**Sleep**			
Omega-3 Fatty acids	Sleep regulation; serotonin activity modulation; melatonin synthesis (DHA)	Emerging evidence especially in the first 1000 days	Long-term, well-designed trials for better sleep outcomes in mother-infants
Vitamin D	Melatonin regulation; VDR activation in sleep–wake regulatory brain regions	Plausible mechanisms and some supportive studies.	High-quality, consistent RCT evidence showing clear, generalizable sleep improvements
Iron	Neurotransmitter synthesis (serotonin, dopamine, norepinephrine); sleep–wake cycle regulation	Strong emerging evidence	High-quality, consistent RCT evidence showing clear, generalizable sleep improvements
Probiotics	Gut–brain axis modulation; neurotransmitter production influencing sleep quality	Strong emerging evidence	Long-term, well-designed trials for better sleep outcomes in mother-infants

Overview of nutrients relevant to the first 1000 days and their identified effects on neurodevelopment, sleep, and gut function. Key knowledge gaps are highlighted to inform priorities for future research across the brain–gut–sleep triad.

## Data Availability

The article does not report new data.

## Abbreviations

The following abbreviations are used in this manuscript:

5-MTHF	5-methyltetrahydrofolate
AS	active sleep
AdCbl	adenosylcobalamin
ASQ	Ages and Stages Questionnaire
AAP	American Academy of Pediatrics
ACOG	American College of Obstetricians and Gynecologists
AA	arachidonic acid
ASD	autism spectrum disorder
ALSPAC	Avon Longitudinal Study of Parents and Children
BSID-III	Bayley Scales of Infant Development-III
BB-12^®^	Bifidobacterium animalis subsp. lactis
BBB	blood–brain barrier
BDNF	brain-derived neurotrophic factor
C-section	cesarean section
CP	cerebral palsy
CSF	cerebrospinal fluid
CFU	colony-forming units
DQ	developmental quotients
DIAMOND	DHA Intake and Measurement of Neural Development Study
DGA	Dietary Guidelines for Americans
DHA	Docosahexaenoic Acid
EPA	eicosapentaenoic acid
EEG	electroencephalography
EFSA	European Food Safety Authority
ERP	event-related potential
Ems	eye movements
GABA	Gamma-Aminobutyric Acid
GID	gestational iodine deficiency
HMOs	human milk oligosaccharides
OHCbl	hydroxocobalamin
HPA	hypothalamic–pituitary–adrenal axis
IUGR	intrauterine growth restriction
IDA	Iron deficiency anemia
KUDOS	Kansas University DHA Outcomes Study
L.	Lactobacillus
LA	linoleic acid
MeCbl	methylcobalamin
MTHFR	methyltetrahydrofolate reductase
MGBA	Microbiota–gut–brain axis
MoBa	Mother, Father and Child Cohort Study
MMN	multiple micronutrients
NAPLAN	National Assessment Program Literacy and Numeracy
NARA II	Neale Analysis of Reading Ability-II
NTDs	neural tube defects
NDDs	neurodevelopmental disorders
NREM	Non-Rapid Eye Movement
PEMT	phosphatidylethanolamine *N*-methyltransferase
PSQI	Pittsburgh Sleep Quality Index
PDX/GOS	polydextrose and galactooligosaccharides
PUFAs	polyunsaturated fatty acid
QS	quiet sleep
REM	Rapid Eye Movement
RBC	red blood cell
RLS	restless leg syndrome
SIgA	secretory immunoglobulin A
SCFAs	short-chain fatty acids
SGA	small-for-gestational-age
SPF	specific-pathogen-free
TSH	thyroid stimulating hormone
USPSTF	United States Preventive Services Task Force
UICs	urinary iodine concentrations
VEPs	visual evoked potentials
WPPSI-III	Wechsler Preschool and Primary Scale of Intelligence-III
WISC-III	Weschler Intelligence Scale for Children-III
ALA	α-linolenic acid
